# Neonatal Tetanus in Vietnam: Comprehensive Intensive Care Support Improves Mortality

**DOI:** 10.1093/jpids/piv059

**Published:** 2015-09-09

**Authors:** Huynh T. Trieu, Inke N. Lubis, Phan T. Qui, Lam M. Yen, Bridget Wills, C. Louise Thwaites, Saraswathy Sabanathan

**Affiliations:** 1 Hospital for Tropical Diseases, Ho Chi Minh City, Vietnam; 2 University of North Sumatera, Jl. Dr. Mansur No. 5, Medan, Indonesia; 3 Oxford University Clinical Research Unit, Hospital for Tropical Diseases, Ho Chi Minh City, Vietnam; 4 Centre for Tropical Medicine and Global Health, Nuffield Department of Medicine, University of Oxford, UK

**Keywords:** autonomic dysfunction, magnesium sulfate, neonatal tetanus

## Abstract

We report a 66% reduction in neonatal tetanus mortality after introducing a new management bundle integrating antibiotic therapy, muscle relaxation and invasive monitoring. The latter allowed rapid detection of autonomic instability which was treated with magnesium sulphate. This is the first report of its use in neonatal tetanus.

## BACKGROUND

Tetanus remains a major preventable cause of neonatal mortality in developing countries. Vietnam commenced an expanded routine immunization schedule for tetanus vaccination of infants in 1981, adding vaccination for pregnant women in 1991, and achieved World Health Organization maternal and neonatal tetanus-elimination status by 2005 [1, 2]. However, neonates with tetanus continue to be admitted to the regional referral center (Hospital for Tropical Diseases, Ho Chi Minh City, Vietnam).

Globally, there were an estimated 49 000 deaths resulting from neonatal tetanus in 2013 [3], and yet there has been a paucity of research into its management; no new therapeutic interventions were described in the last decade [4]. Mortality rates from the disease in low- and middle-income countries remain high (57%–75%) [4]. Autonomic dysfunction is the commonest cause of mortality of adult patients on mechanical ventilation [5], but little is known about its occurrence in neonates.

The Hospital for Tropical Diseases is the designated referral hospital for neonatal tetanus in southern Vietnam (population 40 million). Over the last 20 years, we have observed a notable reduction in the mortality rate of neonates with tetanus at our center, during which time a number of strategies designed to improve care were introduced at the hospital. Facilities for neonatal ventilation became available in 2000 and were associated with a halving of the case-fatality rate from 59% to 32% [6]. A dedicated pediatric intensive care unit, staffed by specially trained doctors and nurses, was established in 2008. In this setting we developed a new neonatal tetanus management bundle (see Table 1). This bundle included components of antibiotics, muscle relaxation, sedation, mechanical ventilation, and routine invasive blood pressure monitoring. In this article we detail the intensive care management bundle, including the first description of the use of magnesium sulfate in neonates with tetanus, and we document the impact of the management bundle in a setting with limited intensive care facilities.

**Table 1.** New Neonatal Tetanus Management Bundle

Equine tetanus antitoxin (1000 IU/kg intramuscularly)^a^
Metronidazole
For infants <7 days old, 15 mg/kg per day orally in divided doses
For infants ≥7 days old, 30 mg/kg per day orally in divided doses
Midazolam infusion (0.1, 0.8 mg/kg per h)^a^
Invasive blood pressure monitoring^a^
Broad-spectrum antibiotics^a^
Cephalosporin (third generation)
Ampicillin
Amikacin
High-flow oxygen via nasal cannula^a^
Indications for intubation: apnea, hypoxemia (Spo_2_ < 90%), uncontrolled spasms^b^
Fentanyl (0.5–2 µg/kg per h)
Magnesium sulfate
Indication: autonomic dysfunction (fluctuation of blood pressure lasting for >1 h, commonly associated with tachycardia and pyrexia)
Dose: loading dose of 50 mg/kg followed by a maintenance infusion of 30–50 mg/kg per h titrated against clinical effect; 6-hourly monitoring of serum magnesium
Pipecuronium
Indication: ongoing spasms while ventilated and on high-dose midazolam
Dose: 0.02–0.05 mg/kg per h

Abbreviation: Spo_2_, peripheral capillary oxygen saturation.

^a^Routine for all patients.

^b^Intubation should not be delayed within 30 minutes of observation.

## METHODS

This was a retrospective study using outcome data from all cases of neonatal tetanus admitted to the Hospital for Tropical Diseases between January 1, 2001, and December 31, 2014. Records for the 7 years after the introduction of the bundle (2008–2014) were compared with records from the 7 years before its implementation (but after mechanical ventilation was available). For comparison of case severity, sufficiently detailed data were available only from 2001 to 2003 and 2010 to 2012; thus, those two 3-year periods were compared.

A retrospective inspection of the hospital records of the 20 patients admitted between January 2010 and December 2012 was performed to obtain more details on the clinical features and severity of these cases. This period was chosen because it allowed time for the new management bundle to be implemented fully and included the period when magnesium sulfate was first introduced.

Before 2008, standard management of neonatal tetanus consisted of administering equine antitoxin, metronidazole, sedation, and benzodiazepines (usually intermittent intravenous diazepam) for spasm control, and if spasms persisted, mechanical ventilation and pipecuronium for muscle relaxation were added.

The new management bundle added the following to standard management (Table 1): (1) first-line therapy with midazolam infusion; (2) early intubation and intermittent positive-pressure ventilation; (3) invasive monitoring of blood pressure; (4) treatment of autonomic dysfunction with magnesium sulfate; and (5) broad-spectrum antibiotics for all infants. The indication for ventilation was cyanosis or spasms not controlled by at least two 0.15 mg/kg boluses of midazolam within 30 minutes. Autonomic dysfunction was diagnosed in the presence of persistent hypertension, tachycardia, or labile blood pressure.

All patient data were entered into a specially designed database and extracted for analysis. Frequencies (%) and medians (interquartile ranges [IQRs]) were used to describe the data. Comparisons between the 2 time periods (before and after introduction of the treatment bundle) for categorical and continuous variables were performed by using Fisher's exact test and the Mann–Whitney *U* test, respectively. All analyses were performed in SPSS version 19 (SPSS, Inc., Chicago, IL). Any *P* value of <.05 was considered statistically significant.

This study was approved by the Scientific and Ethical Committee of the Hospital for Tropical Diseases.

## RESULTS

The records of 91 neonates were obtained; 46 were admitted in the years 2001 to 2007, and 45 were admitted in the years 2008 to 2014. Seven neonates were transferred to other hospitals, and because no further outcome data were available for these infants, they were excluded from the analyses. Therefore, a total of 84 records were used in the initial analysis of mortality, hospitalization, and time from hospitalization to death. Because of the incompleteness of data contained in hospital records between 2004 and 2009, comparisons of prognostic markers were made between data from infants admitted between 2001 and 2003 (34 infants) and 2010 and 2012 (20 infants).

In 2001 to 2007, the in-hospital mortality rate was 36.4% (16 of 44). After implementation of the new management bundle in 2008 to 2014, the mortality rate decreased by 66% to 12.5% (5 of 40; *P* = .013). The times from hospitalization to death were the same in both groups (median, 4.5 days [IQR, 9 days] in 2001–2007 vs 6 days [IQR, 8 days] in 2008–2014; *P* = .86). The durations of hospitalization were similar in survivors (median, 39.0 days [2008–2014] vs 35.5 days [2001–2007]; *P* = .21). There was no difference in indicators of severity between the 2001–2003 and 2010–2012 subgroups as indicated by weight and age (median, 2.80 kg [IQR, 0.5 kg] vs 2.87 kg [IQR, 0.65 kg] [*P* = 0.94] and 8.0 days [IQR, 4 days] vs 8.5 days [IQR, 4.75 days] [*P* = .82], respectively).

In the period of 2010 to 2012, a total of 20 neonates with tetanus were admitted. Eighteen (90%) of them required mechanical ventilation for a median of 32 days (range, 22–50 days). Nine (45%) infants displayed markedly labile blood pressure between days 4 and 11 of illness and were diagnosed with autonomic dysfunction. The median duration of autonomic dysfunction was 10 days (range, 6–23 days). The use of morphine sulfate (0.1, 0.5 mg/kg per h) in the first 3 infants in this series was associated with profound hypotension that required additional inotropic support. To avoid these adverse effects, magnesium sulfate became the first-line therapy. When this regimen was used, no additional therapy was needed to stabilize the blood pressure. Magnesium levels were maintained in the range of 1.0 to 2.5 mmol/L. No clinical manifestations of magnesium toxicity were documented.

In total, 3 (15%) of the 20 patients died. Two patients, aged 13 and 16 days, displayed clinical features of septic shock after 2 and 10 days in the hospital, respectively, although no causative organism was isolated. The third infant died after 2 weeks in the hospital as a result of respiratory failure after an episode of ventilator-associated pneumonia. Other complications in the surviving infants included combined umbilical and inguinal hernias (1 infant) and femoroacetabular dislocation (2 infants). Formal neurodevelopmental assessments were not performed at discharge; however, no gross neurological deficits were noted.

## DISCUSSION

The elimination of neonatal tetanus is defined as <1 case per 1000 live births in every district of a country, and verification involves examination of cases in the most disadvantaged provinces, assuming performance in the other areas will be better. In Vietnam, 3 high-risk provinces were examined, and elimination status was declared in 2005 [1]. Tetanus has not and cannot be eradicated, because the causative bacterium remains widespread in the environment; thus, infants of unvaccinated mothers remain vulnerable, and efforts to improve vaccination coverage and birth hygiene should continue.

The introduction of our management bundle was associated with a significant improvement in the mortality rate. Age and birth weight have been shown to be the most important indicators of disease severity [6, 7], but we were able to compare them only in subgroups of approximately 50% of the total study population; thus, we cannot exclude a confounding effect of severity in the remainder of patients.

Nevertheless, we believe that several factors are likely to have contributed to the recent improved outcome. Referral to a single regional unit enables staff to gain specific experience in managing neonatal tetanus. We found that improved spasm control was achieved by combining continuous midazolam and neuromuscular blockade. Because septicemia is common among neonates with tetanus [8], we believe that the early administration of antibiotics is an important part of routine management.

Finally, invasive blood pressure monitoring enabled early detection of autonomic nervous system dysfunction and timely intervention. We noted that the onset of autonomic dysfunction occurred during the first week of illness, earlier than conventionally described in adults, possibly because of relatively shorter axonal length, although it may also be that the routine use of invasive monitoring facilitated early recognition.

To our knowledge, this report is the first to describe the management of autonomic nervous system dysfunction in neonates with tetanus. Magnesium sulfate is a muscle relaxant that has been used to control tetanus spasms. It also prevents catecholamine release, thus theoretically reducing sympathetic crises [9]. Evidence for the sole use of magnesium sulfate for controlling tetanus spasms is lacking [9, 10], but there is evidence to support its use for autonomic instability in adults [11]. Magnesium has been used in infants for conditions such as persistent pulmonary hypertension of the newborn [12] and is considered safe in this population. Several clinical trials have also assessed its use in suppressing preterm labor, and a recent meta-analysis indicated that is has a neuroprotective effect among surviving premature neonates [13].

The improved survival rate that results from assisted ventilation has highlighted a number of other management challenges for these infants. There was a trend toward increased length of hospital stay, which may have been related to the development of secondary complications. We did not perform long-term follow-up of these infants and therefore cannot exclude the possibility of subsequent neurological disability.

Neonatal tetanus prevention must remain a public health priority [3]; however, future therapeutic options to reduce the need for ventilation and reduce duration of hospitalization merit formal evaluation, especially for use in settings in which intensive care facilities are limited.
